# Catalytic enzymes are active matter

**DOI:** 10.1073/pnas.1814180115

**Published:** 2018-11-01

**Authors:** Ah-Young Jee, Yoon-Kyoung Cho, Steve Granick, Tsvi Tlusty

**Affiliations:** ^a^Center for Soft and Living Matter, Institute for Basic Science, Ulsan 44919, South Korea;; ^b^Department of Biomedical Engineering, Ulsan National Institute of Science and Technology, Ulsan 44919, South Korea;; ^c^Department of Physics, Ulsan National Institute of Science and Technology, Ulsan 44919, South Korea;; ^d^Department of Chemistry, Ulsan National Institute of Science and Technology, Ulsan 44919, South Korea

**Keywords:** enzyme, chemotaxis, active matter, fluorescence correlation spectroscopy, catalytically induced mobility

## Abstract

Using a microscopic theory to analyze experiments, we demonstrate that enzymes are active matter. Superresolution fluorescence measurements—performed across four orders of magnitude of substrate concentration, with emphasis on the biologically relevant regime around or below the Michaelis–Menten constant—show that catalysis boosts the motion of enzymes to be superdiffusive for a few microseconds, enhancing their effective diffusivity over longer timescales. Occurring at the catalytic turnover rate, these fast ballistic leaps maintain direction over a duration limited by rotational diffusion, driving enzymes to execute wormlike trajectories by piconewton forces performing work of a few *k*_*B*_*T* against viscosity. The boosts are more frequent at high substrate concentrations, biasing the trajectories toward substrate-poor regions, thus exhibiting antichemotaxis, demonstrated here experimentally over a wide range of aqueous concentrations. Alternative noncatalytic, passive mechanisms that predict chemotaxis, cross-diffusion, and phoresis, are critically analyzed. We examine the physical interpretation of our findings, speculate on the underlying mechanism, and discuss the avenues they open with biological and technological implications. These findings violate the classical paradigm that chemical reaction and motility are distinct processes, and suggest reaction–motion coupling as a general principle of catalysis.

## Significance

Catalysis and mobility of reactants in fluid are normally thought to be decoupled. Violating this classical paradigm, this paper presents the catalyst laws of motion. Comparing experimental data to the theory presented here, we conclude that part of the free energy released by chemical reaction is channeled into driving catalysts to execute wormlike trajectories by piconewton forces performing work of a few *k*_*B*_*T* against fluid viscosity, where the rotational diffusion rate dictates the trajectory persistence length. This active motion agitates the fluid medium and produces antichemotaxis, the migration of catalyst down the gradient of the reactant concentration. Alternative explanations of enhanced catalyst mobility are examined critically.

Consider a flask of aqueous solution teeming with enzymes during times that they catalyze substrate molecules. The textbook view of the process in the flask, at the nanoscale, is that of molecules bouncing around and whirling as they are continually jolted by thermal fluctuations of the surrounding water (1). This random Brownian motion causes enzymes and substrates to bump into each other. Some collisions end in a substrate specifically bound to the active site of an enzyme, which in turn may convert the substrate into a product molecule. The catalytic reaction is a matter of breaking and forming chemical bonds. Transformation of bonds is orders of magnitude more rapid than Brownian motion, and the separation of timescales leads to the common presumption that there is no cross talk between the chemical activity of catalysts and their spatial mobility.

## Introduction: Leaping Beyond Passive Diffusion

This ingrained view has been challenged by a series of seminal observations showing that enzymes in solution display enhanced diffusivity when they catalyze chemical reactions. This was demonstrated using fluorescence correlation spectroscopy (FCS), first with urease (2), and later with catalase (3) and several other enzymes (4). Enhancement of the diffusion constant, by up to 50%, manifested significant catalytically induced boost of the mobility. Despite concerns about limitation of the experimental technique (5, 6), addressed later in this paper, other experimental methods have confirmed this view. For example, a gradient of substrate induces a predictable, opposite enzyme gradient (7). Nearby inert molecules in solution also exhibit agitated mobility, although they do not participate in the enzymatic reaction (7, 8).

All this signaled a paradigm shift in our understanding of enzymes. Large-scale internal mobility, such as hinge-like rotations, twists, or shear-like sliding, was already linked to the function of enzymes (9–13) in the classical mechanisms of allostery (14) and induced fit (15). However, energetically driven translational motion was considered the exclusive realm of molecular motors (16). In light of the evidence for boosted enzymatic mobility, this distinction appears rather artificial, and one should see enzymes as nanomotors whose dynamic profile influences their function and spatiotemporal organization (3, 17–19).

**Fig. 1.**
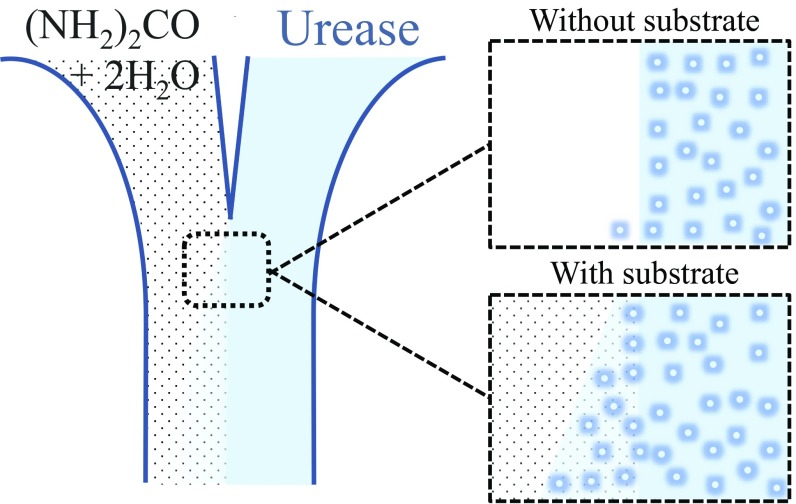
A classical two-channel microfluidic experiment. Injecting substrate solution (and, alternatively, pure buffer solution) from one inlet, and enzyme solution from the other inlet. The enzyme spreads laterally into the substrate stream, much faster than it spreads into an inert buffer stream in a control experiment, and since the enzyme migrates up the substrate concentration gradient, this behavior has been tagged as chemotaxis. However, this migration is directionless chemically enhanced diffusion, and this enhanced diffusion eventually gives rise to antichemotaxis, the formation of an enzyme gradient inverse to that of the substrate. In this paper, we examine and resolve the apparent contradiction.

To examine the taxis of enzymes in substrate gradients, Sen and coworkers (3) developed the now-standard microfluidic assay. In a typical microfluidic measurement, a stream of substrate–enzyme solution is pumped through a microfluidic channel in parallel with a stream of enzyme solution (Fig. 1). They found that the enzyme spreads laterally into the substrate stream, much faster than it spreads into an inert buffer stream in a control experiment. Since the enzyme migrates up the substrate concentration gradient, this behavior was tagged as chemotaxis. However, as we discuss in *Results*, rather than gradient-sensitive chemotaxis, this migration is directionless chemically enhanced diffusion, sometimes termed chemokinesis (20). In addition, quite the opposite, the enhanced diffusion eventually gives rise to antichemotaxis, the formation of an enzyme gradient inverse to that of the substrate. This paper examines and resolves the apparent contradiction, in light of the experimental tests presented here and the mechanisms proposed in the literature.

While the experimental evidence for boosted motion has been mounting in recent years, there remains the challenge to understand the underlying mechanism. Progress on this front was recently made by superresolution fluorescence measurements showing fast leaps of active enzymes (7), proposed as the origin of the enhanced diffusion. Basing on these observations, we formulate here a microscopic theory of catalytically induced motion. Enzymes are self-propelled for durations of a few microseconds by piconewton forces, dissipating energy of a few *k*_*B*_*T* as work against the viscous drag. The enzyme maintains its general direction of motion until rotational diffusion randomizes its orientation. The boosted trajectory is a persistent random walk, curling like a wormlike polymer, ballistic at short times and Brownian with enhanced diffusivity at longer times. The boosts are more frequent at high substrate concentrations, biasing the trajectories toward substrate-poor regions, thus exhibiting antichemotaxis.

In *Results*, we first expound the evidence for ballistic motion and analyze the superresolution FCS data within a microscopic theory of wormlike boosted trajectories. Next, we explain how the boosts make the enzymes self-organize in an antichemotactic pattern with opposite gradients of enzyme and substrate. This is followed by a critical discussion of alternative, noncatalytic passive mechanisms, based on phoresis and cross-diffusion (21, 22), comparing these to our active model in light of the observed superdiffusive mobility and antichemotaxis. We then test the proposed active mechanism by a comprehensive measurement of the enhanced diffusivity of urease over four orders of magnitude of urea and competitive inhibitor concentration, demonstrating catalytically induced enhancement of the mobility in the biologically relevant regime around and below the Michaelis–Menten (MM) constant. *Results* is concluded by control experiments, ruling out possible confounding photophysics effects (6). In *Discussion*, we interpret the present findings, hypothesize about the underlying physical mechanism, and discuss the possible implications.

## Results

### Evidence of Ballistic Motion.

**Fig. 2.**
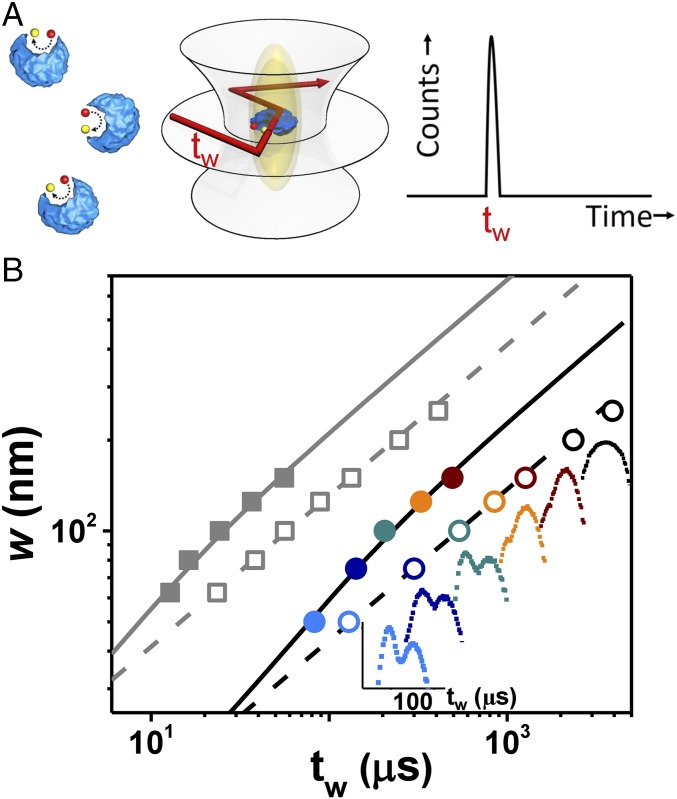
Superresolution STED-FCS measurements compared with theoretical model of substrate-modified enzyme diffusion. (*A*) Urease (blue) hydrolyzes substrate (red) to product. The STED beam narrows the confocal spot to be elliptical. The experimental observables are the transit times (*t*_*w*_) for dye-labeled enzymes to transit the beam waist in the focal plane. The transit times split into two populations, one relatively fast and the other relatively slow. (*B*) Beam waist (*w*) is plotted against transit time, the faster population (filled symbols) and slower populations (empty symbols), for urease in buffer (gray) and urease in the presence of 20% Ficoll (black and colored symbols) during reaction with urea at 1 mM concentration, which presents the advantage of slowing mobility to be more accessible experimentally. For that situation, *Insets* show how the bimodal population distribution changes to unimodal with increasing beam waist; colors are guides to the eye with respective beam waist identified on the *x* axis. In the *Insets*, the scale bar is in microseconds. These data, taken in part from ref. 7, are extended here to a wider range of substrate concentration and compared with the theoretical model proposed in this paper (solid and dashed lines).

Coupling between the activity of enzymes and their boosted mobility takes place on a nanometer length scale inaccessible to diffraction-limited microscopy. Therefore, encouraged by the seminal observations of enhanced diffusion (2–4), in an earlier study we employed superresolution spectroscopy to look into the underlying mechanism at the relevant spatial and temporal scales (7). To this end, we combined stimulated emission depletion (STED) microscopy with FCS (23–25). This allowed us to detect the motion of the enzyme at a resolution limited by the STED beam whose waist *w* was narrowed down to *w* ∼ 50 nm (Fig. 2*A*; data taken in ref. 7, reanalyzed here, and also extended here to a wider range of substrate concentration).

Direct evidence for ballistic motion comes from inspecting the distribution of the transit times, *t*_*w*_, the times it takes an enzyme in solution to cross the interrogatory beam waist within the focal plane. Without substrate present, and hence no chemical reaction, the enzyme transit times are normally distributed, as typical of random Brownian walkers, regardless of the size of the beam waist *w*. Adding substrate radically changes this statistics. Now in the superresolved regime, *w* ≤ 150 nm, the distribution splits increasingly into two modes as the beam narrows: one mode reflecting the expected Fickian Gaussian, and a second faster mode, which becomes increasingly prominent as the beam narrows. In contrast, broader beams (*w* ∼ 200–250 nm) exhibit a single Gaussian mode that can be fitted using standard Brownian dynamics, except that the diffusivity *D*_*E*_ is enhanced by the presence of substrate compared the value *D*_0_ without substrate, *D*_*E*_ > *D*_0_. Similar bimodality was detected when a crowding agent, 20% Ficoll, was added to the solution, increasing about 10-fold (to ∼10 cP) the effective viscosity. For the narrowest STED beam, detailed inspection of the fast superdiffusive mode showed that it was better fitted by a most probable time with an exponential tail, unlike the Gaussian diffusive mode. This implies that the kinematics of active enzymes and random Brownian particles are fundamentally different at this nanometer length scale. This qualitative difference goes beyond merely an enhanced value of the diffusion coefficient.

Deviations of catalytically active enzymes from Fickian dynamics become more evident when one scales the fast peak transit time $t_{w}^{\text{fast}}$ by the beam waist *w*. The data start to deviate from the square scaling of Brownian motion at high *w* and gradually approach linear scaling for the smallest length scales *w* (Fig. 2*B*). In contrast, the slow peak transit time scales as $t_{w}^{\text{slow}}∼w^{2}$, not only in the absence of substrate but also with substrate present. This anomalous behavior of the fast component suggests the possibility that, at the short time and length scales probed by this superresolution experiment, enzymes move ballistically. Their trajectories should include short catalytically induced impulses, or “leaps,” punctuated by durations of Brownian diffusion. This picture is consistent with the observed transit times, $t_{w}^{\text{fast}}∼8\mu \text{s}$ for a beam waist of *w* = 50 nm, which is shorter than the minimal turnover time, $k_{\text{cat}}^{-1}∼17\mu \text{s}$. Here, $k_{\text{cat}}∼6\cdot 10^{4}s^{-1}$ is the turnover number of a urease hexamer at 25 °C (26, 27), indicating that, on average, one monomer in the urease hexamer will be active during an interval of $k_{\text{cat}}^{-1}∼17\mu \text{s}$.

As to why the family of fast transit times approaches linear ballistic scaling gradually as *w* is reduced, we suggest that rotational diffusion gradually deflects enzymes during their ballistic motion, eventually causing their trajectories to lose the original direction. This loss of directed motion occurs at timescales $\tau_{R}$ inversely proportional to the rotational diffusion coefficient, $\tau_{R}∼D_{R}^{-1}=4\pi \eta R^{3}/k_{B}T$, where *η* is the viscosity of the medium, and *R* is the effective hydrodynamic radius. The rotational timescale for the urease hexamer [*R* ∼ 6–12 nm (28)] is in the range *τ*_*R*_ ∼ 1–6 μs, implying that the STED beam accesses the transition to the ballistic regime, as manifested by the deviation from diffusive scaling.

### Wormlike Trajectories.

The nondiffusive dynamics detected at the nanometer length scales motivated us to examine and to quantify how the proposed ballistic mechanism affects the mobility and the self-organization of catalytic systems. The enzyme can be seen as a nanoswimmer that is boosted by rapid stochastic impulses fueled by its chemical activity. Boosts occur stochastically at the average turnover rate, and in between the enzyme diffuses passively by thermal Brownian motion. In this low-Reynolds regime of nanometric self-propulsion, $\text{Re}∼10^{-5},$ one can disregard inertia and assume overdamped Langevin dynamics for **v**(*t*) the enzyme velocity. Hence, $\gamma v\left(t\right)=f_{T}\left(t\right)+f_{B}\left(t\right)$, where *γ* is the coefficient of the friction exerted on the enzyme, resisting two types of stochastic forces, the standard uncorrelated thermal force $f_{T}$ and an episodic catalytically driven boost $f_{B}$. For simplicity, we consider a boost whose amplitude $f_{B}$ is constant during a boost time $\tau_{B}$, and whose orientation becomes uncorrelated by rotational diffusion at a timescale $\tau_{R}$.

From the fluctuation–dissipation theorem (29, 30) (*Methods*), we find the enhanced diffusion coefficient, $D_{E}$:

$$D_{E}=D_{0}+D_{B}=D_{0}+\frac{V}{3}{\left(\frac{f_{B}\tau_{R}}{\gamma }\right)}^{2}\left(e^{-\tau_{B}/\tau_{R}}-1+\frac{\tau_{B}}{\tau_{R}}\right).$$ [1]

The passive thermal diffusivity is $D_{0}=k_{B}T/\gamma$ and the boosted one *D*_*B*_ is proportional to the enzyme catalysis rate V, which is governed by standard MM kinetics, $V=k_{\text{cat}}c/\left(K_{M}+c\right)$, where $k_{\text{cat}}$ is the turnover number, $K_{M}$ is the MM constant, and *c* is the substrate concentration. Eq. **1** has two asymptotic regimes. First, bursts too short for the enzyme to lose orientation, $\tau_{B}\ll \tau_{R}$, yield a boosted diffusivity, $D_{B}\approx Vl_{B}^{2}/6$, where $l_{B}=v_{B}\tau_{B}$ is the distance traversed by the boosted enzyme at a velocity $v_{B}=f_{B}/\gamma$. At the other extreme, $\tau_{B}\gg \tau_{R}$, and we find $D_{B}\approx \left(\tau_{R}/\tau_{B}\right)Vl_{B}^{2}/3$. The enzymes we studied experimentally are in the intermediate regime, $\tau_{B}∼\tau_{R}$. Here, one can approximate the boosted diffusivity as $D_{B}∼Vl_{B}^{2}/\left(3e\right)$.

To link the boost to the energetics of the enzymatic reaction, we estimate the work performed against the drag force during the boost (in units of $k_{B}T$) as $b=f_{B}v_{B}\tau_{B}/k_{B}T.$ Likewise, the energy dissipated during the rotational timescale $\tau_{R}$ is $p=f_{B}v_{B}\tau_{R}/k_{B}T$, and the dimensionless number *p* measures how far the system is driven from equilibrium. With these definitions, the time ratio becomes $\tau_{B}/\tau_{R}=b/p$, and the enhanced diffusion is expressed in energetic terms as follows:

$$D_{E}=D_{0}+D_{B}=D_{0}+\frac{1}{3}D_{0}V\tau_{R}\cdot p\left(e^{-b/p}-1+\frac{b}{p}\right).$$ [2]

The boosted motion enhances the diffusivity by a factor proportional to the number of substrates converted into products during the rotational timescale, $D_{B}/D_{0}\propto V\tau_{R}$. Approximating the rotational diffusion and friction by the Stokesian expressions for spherical bodies, $\tau_{R}=4\pi \eta R^{3}/k_{B}T$ and $\gamma =k_{B}T/D_{0}=6\pi \eta R$, we find that the boosted diffusion constant scales like the squared size of the enzyme complex times its enzymatic activity $D_{B}∼VR^{2}$. Hence, the ballistic motion is most noticeable for high turnover enzymes that self-assemble in complexes of large hydrodynamic radius. Ballistic motion may occur also in smaller or low turnover enzymes, but it would be hard to probe it with the present FCS method.

In the STED-FCS measurements (Fig. 2), the boost and the rotation times are similar, $\tau_{B}/\tau_{R}=b/p∼1$. Trajectories in this regime begin ballistically and then, wobbling by the rotational diffusion, stray increasingly from the original orientation. The consequent scaling of the mean-square displacement (MSD) ${\langle r^{2}\rangle }_{\text{fast}}$ of the fast transit peaks is in the intermediate regime between diffusive and ballistic,

$${\langle r{\left(t\right)}^{2}\rangle }_{\text{fast}}=6D_{0}t+2D_{0}\tau_{R}\cdot p\left(e^{-t/\tau_{R}}-1+\frac{t}{\tau_{R}}\right).$$ [3]

The first linear term, $6D_{o}t={\left(2R\right)}^{2}\left(t/\tau_{R}\right)$, is the contribution of thermal diffusion, demonstrating that in the absence of substrate the enzyme will lose its original orientation after traversing about one diameter. The boost contribution in [**3**] is the term proportional to *p* that maintains the enzyme oriented over longer distances (*Methods*). Thermal rotational fluctuations during the boost mask the square scaling, ${\langle r^{2}\rangle }_{\text{fast}}∼p\left(D_{o}/\tau_{R}\right)t^{2}={\left(v_{B}t\right)}^{2}$, of pure ballistic motion.

The MSD of the boosted enzyme brings to mind that of a wormlike polymer (31, 32): The trajectory of the persistent random walk (33–37) is equivalent to the momentary configuration of the wormlike chain for which the ratio $t/\tau_{R}$ in Eq. **3** would be replaced by the polymer length measured in persistence length units. The boosted enzyme changes its direction in the same fashion that the director along a wormlike polymer loses it orientation, with the temporal correlation of the enzyme’s direction decaying exponentially just like the spatial correlation of the polymer’s director. In the language of this polymer analogy, the observed trajectories $l_{B}=v_{B}\tau_{B}$ are not much longer than the “persistence length,” $l_{R}=v_{B}\tau_{R}$. Possible physical mechanisms underlying the phenomenology of boosted motion that motivates the proposed model, Eqs. **2** and **3**, are examined in *Discussion*.

Despite the simplicity of the model (Eqs. **1**–**3**), in particular the presumption of a constant boost force, fits of the measured transit times of the fast component are in excellent agreement with Eq. **3**. In this comparison (*Methods*), we use the same parameters to fit both the fast and the slow diffusive components such that the slow component scales as a 3D Brownian walk with enhanced diffusivity $D_{E}$ given by Eqs. **1** and **2**, so that ${\langle r^{2}\rangle }_{\text{slow}}=6D_{E}t$. Specifically, the estimated force $f_{B}∼1\text{pN}$ acts along a trajectory $l_{B}∼43\text{nm}$ for a duration $\tau_{B}∼6\mu \text{s}$, boosting the urease at a velocity $v_{B}∼7\text{nm}/µ\text{s}$ and dissipating an energy $b∼10k_{B}T$ against the viscous drag, so the work required for the boost does not exceed the typical energy scales of enzymatic reactions. The rotational diffusion time is $\tau_{R}∼6\mu \text{s}$, and the dimensionless measure p∼9 signifies that the system is driven relatively far from equilibrium. The enhanced diffusion estimated from Eqs. **1** and **2** is $D_{B}∼10{\mu m}^{2}/\text{s}$ for $V∼k_{\text{cat}}$, in line with the observed effect.

When surrounded by the crowding agent Ficoll which enhances the solution viscosity by a factor of 10, urease mobility becomes 10-fold slower, and comparison of the model to data shows that enzymes now move at velocity $v_{B}∼0.8\text{nm}/µ\text{s}$ with rotational diffusion time $\tau_{R}∼57\mu \text{s}$. The boost persists longer, for $\tau_{B}∼19\mu \text{s}$, and the increased viscosity shortens the trajectory to $l_{B}∼14\text{nm}$, dissipating less energy, $b∼4k_{B}T$. Still, the boost force $f_{B}∼1.1\text{pN}$ remains similar to that measured in the absence of Ficoll, and so is the out-of-equilibrium parameter p∼11, supporting our hypothesis of chemically boosted propulsion. Interestingly, the boost time with Ficoll present is comparable to the minimum catalysis time, $1/k_{\text{cat}}∼17\mu \text{s}$ for the urease hexamer (26, 27). This implies that, when saturated with substrate, the hexamer is being boosted most of the time.

The conclusion that fast enzymes ballistically crossed the interrogatory experimental windows holds regardless of the enzyme concentration. A fast component with similar transit times was observed even when the substrate concentration was tenfold smaller, but as fewer enzymes were active in this case at any given moment, the abundance fraction of fast enzymes decreased by this same factor of 10 and the fast component was correspondingly less discernible in the data. For the different enzymes cholinesterase (AChE), a similar bimodal transit time distribution was confirmed in the presence of its substrate, acetylcholine (ACh) (7). Comparing that data to our model, we find that the AChE boost persists for $\tau_{B}∼17\mu \text{s}$, exerting a force $f_{B}∼0.4\text{pN}$ along a trajectory $l_{B}∼35\text{nm}$, moving the enzyme at a velocity $v_{B}∼2\text{nm}/\mu \text{s}$ and dissipating energy of $b∼3k_{B}T$ as work against the viscous drag. The rotational diffusion time of the AChE enzyme is $\tau_{R}∼46\mu \text{s}$ (38–40) and the out-of-equilibrium measure is p∼9. The similarity of physical scales extracted from the ballistic kinematics of two dissimilar enzymes further establishes the link between enhanced diffusion and catalytically induced ballistic motion.

The enhanced diffusion grows with the reaction rate as the saturation curve of the MM kinetics, $D_{B}\propto V\left(c\right)=k_{\text{cat}}c/\left(K_{M}+c\right)$, as noted previously by several authors (3, 4, 21, 41, 42), although a square root law of Fickian diffusion was sometimes stated, for urease (3). To explain the data, Riedel et al. (4) suggested the intriguing scenario that the boost originates from sudden release of reaction enthalpy, inducing an asymmetric pressure pulse that displaces the enzyme. However, the timescale of this proposed “chemoacoustic” mechanism is that of pressure equilibration, roughly the time it takes a sound wave to cross through the enzyme, ∼10 ps, much faster than the boost time, $\tau_{B}∼10\;\mathrm{\mu s}$. Recently, noncatalytic mechanisms have been proposed (21, 42). In the context of the antichemotaxis demonstrated by experiments (7), our later discussion assesses these passive mechanisms.

The energetics of the boosts assumed in our model (Eqs. **2** and **3**) are consistent with enzyme biochemistry and with the measured enhancement of the diffusivity. The overall picture suggested is of Brownian diffusion of the enzyme, punctuated by impulsive directional leaps that transpire at times when the enzyme is catalyzing. In the overdamped low-Reynolds regime of nanoswimming, momentum transfer from ejection of reaction products cannot explain the boost. The inertial relaxation time of the enzyme is $\tau_{I}=m/\gamma ∼5\;\text{ps}$, implying that the boost requires a force impulse sustaining throughout the boost time, $\tau_{B}∼10\;\mathrm{\mu s}$, or alternatively a series of shorter impulses. During the boost period, the enzyme traverses a distance of 2–5 enzyme length dimensions, and dissipates an energy of ∼1–2 $k_{B}T$ per traversed radius to maintain this directional motion, which reduces the entropy. The work should be provided by ΔG=ΔH−TΔS, the Gibbs free energy released by the reaction, such that enthalpy change ΔH is modulated by the entropic contribution −TΔS. An ongoing discussion examines the question whether enhanced diffusivity requires exothermic reaction, ΔH≤0 (4, 21, 22, 42). However, additional entropy provided by product release may allow for endothermic boosts (42).

### Antichemotaxis.

Bacteria, when they forage for food, use an evolved “run-and-tumble” algorithm to migrate up a gradient of nutrient, without the need to sense the spatial gradient (43–46). Their strategy to accomplish this is progressive reduction of tumbling frequency with higher nutrient concentrations, thereby biasing the random walk toward regions rich in food, a mechanism termed “chemotaxis.” Generalizing this concept to the realm of enzymes, we observe that the frequency of catalytically induced boosted motion also grows with the turnover rate. Concentration dependence according to the MM reaction kinetics, V(c), might be perceived to put forward the possibility of analogous catalytically driven taxis at the nanoscale. Indeed, the seminal measurements by Sen and coworkers (3) were interpreted to show enhanced mobility of enzymes toward their substrate compared with enzymes diffusing thermally in the same buffer but without substrate, thus suggesting the possibility of enzymatic chemotaxis. However, as we discuss below, the motilities of enzymes and bacteria are based on opposite algorithms. While bacteria tumble less frequently in nutrient-rich locations, enzymes leap more frequently when provided with more substrate. The resulting directions of taxis are opposite: chemotaxis (for bacteria) and antichemotaxis (for enzymes).

**Fig. 3.**
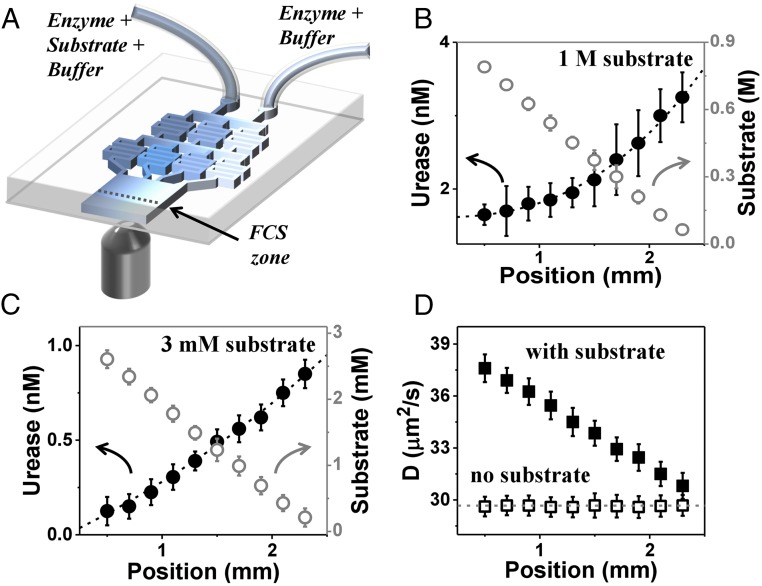
Microfluidic experiment demonstrating antichemotaxis when urease is catalytically active. (*A*) Schematic diagram of the microfluidic chip. The enzyme–substrate–buffer (E+S+B) enters one inlet, and the substrate-free enzyme solution in buffer (E+B) enters another, producing constant enzyme concentration across the channel but a linear gradient of its substrate. (*B*) Experiments at high substrate concentration: The urease concentration and substrate concentration in microfluidic chip are 5 nM and 1 M, respectively. Urease concentration extracted from FCS autocorrelation fitting and calibrated urea concentration (empty circles) are plotted against position across the channel with error bars showing SD of five repeated measurements. (*C*) Experiments at low substrate concentration: The urease concentration and substrate concentration at the chip inlet are 1 nM and 3 mM, respectively. (*D*) The enzyme diffusion coefficient (*D*) extracted from FCS autocorrelation fitting with a diffraction-limited spot size, plotted against position in the channel, in the presence (filled squares) and absence (open squares) of substrate, for the case of *B* (1 M substrate).

To test the antichemotaxis prediction experimentally, we generated a steady-state gradient of substrate concentration and measured the induced spatial pattern of enzyme concentration (Fig. 3*A*) using methods described previously (7), but with a much wider range of substrate concentration and with enzyme concentration low enough to avoid significant consumption of the substrate. At the input of this microfluidic device, the enzyme was pumped with a uniform concentration across the microfluidic channel, but as it flowed downstream and approached steady state, it developed a concentration profile whose gradient opposed that of the substrate and the enhanced diffusion coefficient (2–4) (Fig. 3*B*). Thus, enzymes performed antichemotaxis, migrating away from substrate-rich regions. In the absence of substrate, both enzyme concentration and diffusivity remained uniform along the channel. Therefore, antichemotaxis was linked to the presence of substrate, presumably to its catalysis.

How enhanced mobility generates spatiotemporal organization of catalytic systems follows from considering a generalized Fick’s law for the current of the enzyme concentration ρ,

$$J_{E}=-\nabla \left[D_{E}\left(c\left(r,t\right)\right)\cdot \rho \left(r,t\right)\right]\;,$$ [4]

where the concentration-dependent mobility is $D_{E}\left(c\right)=D_{0}+D_{B}=D_{0}+\alpha V\left(c\right)$, with the enzyme characteristic $\alpha ∼l_{B}^{2}$ defined by Eq. **1**. Fick’s law [**4**] follows directly from the isotropic Langevin dynamics lacking drift (47–49), and the consequent diffusion equation is as follows:

$$\frac{\partial \rho \left(r,t\right)}{\partial t}=-\nabla J_{E}=\nabla^{2}\left[D_{E}\left(c\left(r,t\right)\right)\cdot \rho \left(r,t\right)\right]\;\cdot$$ [5]

In general, this dynamics would be completed by a reaction–diffusion equation describing the random walk of the substrate molecules and their consumption by the enzyme. However, we designed our experiments such that the linear gradient of the enzyme changed only slightly, owing to continuous pumping at the input. Assuming that the profile reached steady state and depends only on the coordinate *x* across the channel, Eq. **5** disregards the negligible contribution of downstream current. In this regime, the overall current vanishes, $J_{E}=-\nabla \left[\rho \left(x\right)D_{E}\left(c\right)\right]=0.$ The resulting inverse relation, $\rho \left(x\right)\propto 1/D_{E}\left(c\right)=1/\left[D_{0}+\alpha V\left(c\left(x\right)\right)\right]$, implies antichemotaxis of the enzyme. Inclusion of downstream convection would modify this conclusion, but only quantitatively: at steady state, the gradients of substrate and enzyme are opposite.

In the experiment, drifting down this microfluidic channel for about $t_{\text{channel}}∼\;50\;\text{s},$ an enzyme reaches the measurement line after turning over $n_{\text{s}}∼V\left(c\right)t_{\text{channel}}$ substrate molecules (Fig. 3*A*). At substrate concentration *c* = 3 mM, each enzyme consumes $n_{\text{s}}∼6\cdot 10^{5}$ substrates before reaching the measurement line. With ρ=1 nM of enzyme, there are $3\cdot 10^{6}$ substrates per enzyme, implying that less than 20% of the substrate is consumed. Therefore, the substrate gradient at the detection line is only slightly shallower than the original gradient at the channel’s input. With most substrate unconsumed, the turnover rate remains high downstream, resulting in substantial enhancement of the diffusivity. All of our observations, including one at the high concentration regime, c=1 M, exhibit antichemotaxis such that the enzyme and substrate gradients are opposite in sign. While it is true that to attain exact steady state would require longer times than were practical to wait owing to the consumption of substrate, we emphasize that over a channel width of ∼1–2 mm, gradients of enzyme and substrate were opposite.

Our measurements of enhanced mobility agree with earlier measurements of enhanced enzymatic mobility in the presence of substrate (2, 3) (Fig. 1), except that we observe antichemotactic migration of enzyme away from the substrate-rich region, not chemotaxis as these studies reported. The apparent contradiction is a matter of how one interprets the data, which itself is not in doubt. The reported microfluidic measurements started without enzyme in the substrate current (Fig. 1). Spreading of enzyme was induced by the normal tendency of Brownian diffusion to even out concentration gradients. Spreading into the substrate stream was faster than into a buffer stream thanks to the enhanced diffusion constant, governed by the local concentration of the substrate, not its gradient.

An instructive analogy is the expansion of gas from a cold vessel into a hot vacuum chamber. Gas will expand faster when the vacuum chamber is hotter, but temperature itself drives the expansion, not the temperature gradient. Likewise, enhanced spreading of the enzyme originates in a directionless mechanism rather than gradient-sensitive chemotaxis. This becomes evident when the system is allowed to approach a steady state. In the gas expansion analogy, the steady-state gas density in the hot chamber will be less than in the cold vessel, a phenomenon one may call “antithermotaxis.” To conclude, the sign of taxis is determined by the migration tendency of an enzyme molecule, as measured after it has had time to explore the substrate gradient. For this reason, our experiment starts with a uniform enzyme profile and this was observed to evolve an opposing gradient owing to the antichemotactic tendency. A similar effect was recently modeled in spherical geometries by Weistuch and Pressé (41), who termed the separation of enzyme and substrate “repulsion.”

The observed antichemotaxis demonstrates that enzymes can use the energy dissipated in chemical reactions to increase their spatial order and reduce the entropy by self-organizing into these nonuniform patterns. Because the rate of ballistic boosts increases with substrate concentration, the enhanced mobility drives enzymes to accumulate in substrate-poor regions where their mobility is slower. Thus, antichemotaxis spatially homogenizes the rate of catalysis by inverting the gradients of enzyme and substrate, and hence may be useful for optimizing the yield of reaction networks in the cell.

### Noncatalytic Passive Mechanisms That Enhance Mobility.

The discovery that enzymes move faster in the presence of their substrates (2–4) inspired a series of theoretical works suggesting underlying mechanisms (4, 21, 22, 42). Recently, noncatalytic passive mechanisms have been proposed (21, 42) in which the MM-like concentration dependence originates from an equilibrium binding curve. To examine these models, we split the generalized Fick’s law [**4**] into a contribution from directionless enhanced diffusion, and a gradient-sensitive taxis term (46):

$$J_{E}=-D_{E}\nabla \rho -\left(\frac{\partial D_{E}}{\partial c}\nabla c\right)\cdot \rho =-D_{E}\nabla \rho +\left(\sigma_{B}\frac{\nabla c}{c}\right)\cdot \rho \;,$$ [6]

where the taxis diffusivity $\sigma_{B}$ is

$$\sigma_{B}=-c\;\frac{\partial D_{E}}{\partial c}=-\left(\alpha k_{\text{cat}}\right)\cdot \frac{K_{M}c}{{\left(K_{M}+c\right)}^{2}}\;.$$ [7]

The taxis is up or down the substrate gradient as determined by the sign of $\sigma_{B}$; chemotaxis if $\sigma_{B}>0$ and antichemotaxis if $\sigma_{B}<0$. For the catalytically enhanced diffusion of our model, $\sigma_{B}=-\alpha c\left(\partial V/\partial c\right)<0,$ since both $\alpha ∼l_{B}^{2}$ and ∂V/∂c are always positive, implying antichemotaxis at all conditions. The conclusion is not limited to MM kinetics and relies only on the natural assumption that the catalytic rate V(c) increases with substrate concentration, and is valid also for collective enzyme kinetics, such as the Hill curve.

Based on experimental evidence (50, 51) and theoretical predictions (52), Zhao et al. (22) proposed ligand binding as the origin of what they considered to be chemotactic activity of enzymes. In this scenario, “cross-diffusion” is induced by preferential attraction or repulsion of the ligands, which becomes asymmetric in a ligand gradient, thereby producing a net driving force. The resulting enzyme current is similar to Eq. **6**, $J_{\text{x}}=-D\nabla \rho +\left(\sigma_{\text{x}}\nabla c/c\right)\cdot \rho$, where the cross-diffusion diffusivity is $\sigma_{\text{x}}=D\cdot c/\left(K_{D}+c\right)$, with the dissociation constant $K_{D}∼K_{M}$. Cross terms are known to form inhomogeneous patterns in reaction–diffusion systems (51, 53) and were suggested as the mechanism underlying the “focusing” of hexokinase toward its substrate d-glucose (15), in line with the positive sign of $\sigma_{\text{x}}$ indicating chemotaxis. For the same reason, cross-diffusion cannot explain the observed antichemotaxis in our experiment that requires a negative $\sigma_{B}$.

A binding-phoresis mechanism in the same spirit was introduced by Agudo-Canalejo et al. (21), who separated between contributions from specific and nonspecific short-range interactions with the substrate. Nonspecific binding gives rise to phoretic forces with diffusivity $\sigma_{\text{ph}}=\left(6\pi R\lambda^{2}c\right)D_{0}$, where the small Derjaguin length, ∼1–8 Å, implies that phoresis becomes noticeable only when the concentration is high, *c* ∼ 100 mM, orders of magnitude above the biologically relevant regime. The contribution of specific binding (21) arises from a proposed change in the diffusivity of enzymes when bound to substrates, $D_{\text{bound}}=D_{0}+\Delta D$. The overall diffusivity is the average over free and specifically bound states according to the binding curve, $D_{\text{sb}}\left(c\right)=D_{0}+\Delta D\cdot c/\left(K_{M}+c\right)$. One may intuitively expect that a bound enzyme diffuses more slowly than a free one, ΔD<0, since it is larger and more massive, reducing the overall diffusion $D_{\text{sb}}\left(c\right)<D_{0}$. Intriguingly, Agudo-Canalejo et al. suggest that binding may nonetheless make the enzyme diffuse faster, ΔD>0, by coupling to its internal degrees of freedom, or even by reducing its effective hydrodynamic radius and friction coefficient (42), implying enhanced diffusivity, $D_{\text{sb}}\left(c\right)>D_{0}$. It is not clear whether such effects are generic, or explain the enhanced diffusion that is likewise observed for small organometallic catalysts (54), where there is little coupling to internal degrees of freedom, and one expects an increased hydrodynamic radius. At any rate, the proposed scenario of binding-enhanced diffusivity cannot explain the observed ballistic motion (7) that remains directional over distances $l_{B}∼40\;\text{nm}$ and durations *τ*_*B*_ ∼ 10 μs. Reducing the friction coefficient γ by, say 30%, would indeed boost the diffusivity $D=k_{B}T/\gamma$ by this same factor, but the velocity correlation time and distance will remain in the range $\tau_{I}=m/\gamma ∼5\;\text{ps}$ and $l_{I}=\sqrt{6D_{0}\tau_{I}}∼0.3\;Å$. Both are orders of magnitude less than the observed boost.

The concentration dependence of the binding-induced diffusivity gives rise to the generalized Fick’s law [**4**], $J_{\text{sb}}=-\nabla \left(D_{\text{sb}}\cdot \rho \right)=-D_{\text{sb}}\left(\text{c}\right)\nabla \rho +\left(\sigma_{\text{sb}}\nabla c/c\right)\cdot \rho$, derived from a specific two-state model (21), and previously from generic isotropy (7). The first term is the directionless enhanced or reduced diffusion, and the second is a chemotactic diffusivity similar to Eq. **7**, $\sigma_{\text{sb}}=-c\left(\partial D_{\text{sb}}/\partial c\right)=-\Delta D\cdot K_{M}c/{\left(K_{M}+c\right)}^{2}$. If binding slows the enzyme, ΔD<0, then $\sigma_{\text{sb}}>0$, and chemotaxis is in the direction of the substrate gradient. Hypothesizing a faster bound enzyme, ΔD<0, implies $\sigma_{\text{sb}}<0$ and antichemotaxis. As the theoretical arguments related the diffusivity change ΔD to changes in the enzyme’s elastic moduli due to binding (42), the significant diffusivity enhancements observed so far would require corresponding major alterations of these moduli. In comparison, our phenomenological model relates enhanced diffusivity to the kinetic rate and observed ballistic kinematics, $\Delta D∼Vl_{B}^{2}/3e∼10{\;\mu m}^{2}/\text{s}$.

Unlike our catalytically induced boost mechanism, the cross-diffusion (22) and phoresis-binding (21) mechanisms are passive and require no energy consumption. Their apparent MM-like concentration dependence originates from the binding curve of the substrate, not its catalysis. Consequently, any specific binding to the enzyme, including binding of a competitive inhibitor, is predicted to similarly enhance the diffusivity. Moreover, specific binding to noncatalytic macromolecules is expected to have the same effect. So far, one such demonstration has been published (42), where the diffusivity of the enzyme aldolase appeared to increase, above the noise level, in the presence of the competitive inhibitor pyrophosphate (PPi). However, it was recently proposed that the apparent enhanced diffusivity might be the outcome of dissociating aldolase tetramers (6, 55). The passive hypothesis may be strengthened by further observations.

### Bimodal Concentration Dependence of Enhanced Diffusion.

It appears that boosted antichemotaxis, and the proposed cross-diffusion or phoresis, belong to different concentration regimes. The boost mechanism becomes significant already at $c∼K_{M}$, typically in the ∼0.1–3 mM range (56), while focusing and cross-diffusion were imputed when the concentration was significantly higher, ∼50–100 mM (22). Indeed, we are not aware of any report of positive chemotaxis at concentrations $c∼K_{M}$. The biologically relevant operation regime of enzymes, however, is around and below their MM constants, $c∼K_{M}$ (1, 57), which are widely distributed around a median of $K_{M}∼0.13\;\text{mM}$ (56). At higher substrate concentrations, enzymes would become inefficient metabolic bottlenecks with a flat response curve.

**Fig. 4.**
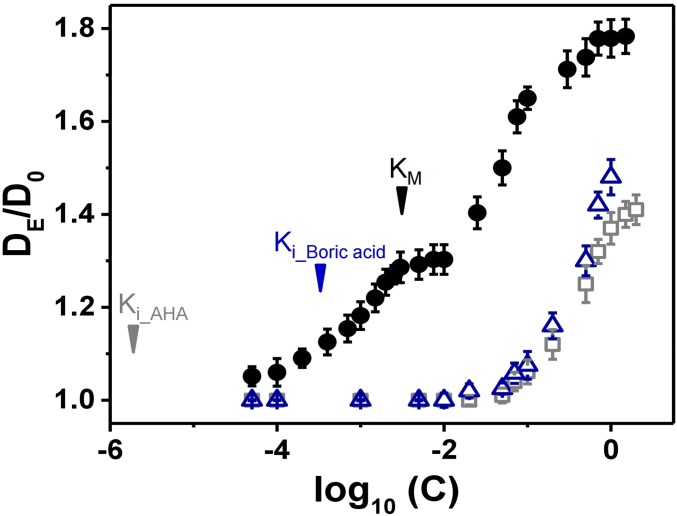
In the biologically relevant regime of concentrations at and below the MM constant *K*_M_ (3 mM) indicated on this graph, enzyme diffusion is enhanced by the presence of substrate but not of enzyme inhibitor. However, enzyme diffusion is enhanced by both of them at higher concentration *c* > 100 mM. Here, for 10 nM urease, the normalized enhanced diffusion *D*_E_/*D*_0_ (*D*_o_ is diffusion in pure buffer) is plotted against substrate concentration (black symbols) and inhibitor concentration (blue open symbols, boric acid; gray open symbols, acetic hydroxamic acid), each of them varied by four orders of magnitude. The inhibition constant (*K*_i_) of boric acid and acetic hydroxamic acid, 0.33 mM and 2 µM, respectively, are also indicated on the graph.

To test our hypothesis of distinct physical mechanisms, we mapped out the diffusivity $D_{E}$ of urease across four orders of magnitude of urea concentration, *c* = 100 μM to 1 M (Fig. 4). Enhanced diffusion was detected starting at the smallest concentrations and saturated above the MM constant, $c∼K_{M}∼3\;\text{mM}$. We observed a plateau of diffusivity, $D_{E}∼1.3D_{0}$ up to *c* ∼ 50 mM, above which $D_{E}$ rose again, approaching a second plateau, $D_{E}∼1.8D_{0}$ at *c* ∼ 1 M. The observation of two plateaus supports the two-mechanism hypothesis, and we therefore repeated the urease diffusivity assay, now in the presence of two competitive inhibitors, boric acid and acetohydroxamic acid (AHA) (58, 59) (experimental). Ballistic motion was never observed, regardless of the inhibitor concentration. Neither inhibitor enhanced diffusivity except at concentrations c>30 mM. At these higher concentrations, enhanced diffusion became noticeable and exhibited similar saturation curves, parallel on the concentration scale to that of urea over this same range of *c*, but shifted downward on the vertical scale, indicating lower magnitudes of enhanced diffusion. The comparison suggests that the apparent diffusivity of urease is a sum of two independent mechanisms, catalytically induced enhancement and a passive mechanism whose onset is at high substrate concentrations. At biologically relevant concentrations, $c\leq K_{M}∼3\;\text{mM}$, only the active mechanism is relevant. It is noteworthy that the inhibitor constants, $K_{I}∼0.33\;\text{mM}$ for boric acid and 2 μM for AHA, are smaller even than *K*_M_, and accordingly appear irrelevant to the passive mechanism, which is noticeable only at much high concentrations. The apparent enhancement in this concentration regime may also originate from dissociation of enzyme complexes into smaller subunits (6, 55).

To conclude, there are two main classes of theoretical explanations for the enhanced diffusion and antichemotaxis observed in experiments: catalytic mechanisms, relying on energy provided by substrate turnover (4, 7), and noncatalytic passive mechanisms in which short-range interactions with substrate molecules enhance the rate of enzyme mobility (21, 22). The directional ballistic motion detected in STED-FCS measurement cannot be explained by the passive mechanisms as it entails energy dissipation. Furthermore, in the absence of substrate, we observed no fast directional component. The proposed passive mechanisms of phoresis and cross-diffusion may be significant only at high concentrations, *c* ∼ 100 mM and above, and enzyme dissociation into smaller subunits (6, 55) may also contribute.

In the biologically relevant concentration regime, *c* ∼ *K*_M_, we observed enhanced diffusion and antichemotaxis and explain both as direct consequences of the ballistic motion. In contrast, the alternative phoresis and cross-diffusion hypotheses predict positive chemotaxis regardless of concentration. Another hypothesized passive mechanism is that diffusion of bound enzymes may be enhanced by coupling to the internal degrees of freedom and resulting reduction of the hydrodynamic friction. This intriguing mechanism could be further tested in comparison with our proposed active mechanism by detecting antichemotaxis and enhanced diffusion of enzymes in the presence of competitive inhibitor at low concentrations. However, such passively enhanced diffusion was not observed in our urea competitor assays in the biologically relevant regime around and below the MM constant (Fig. 4).

### Further Control Experiments.

**Fig. 5.**
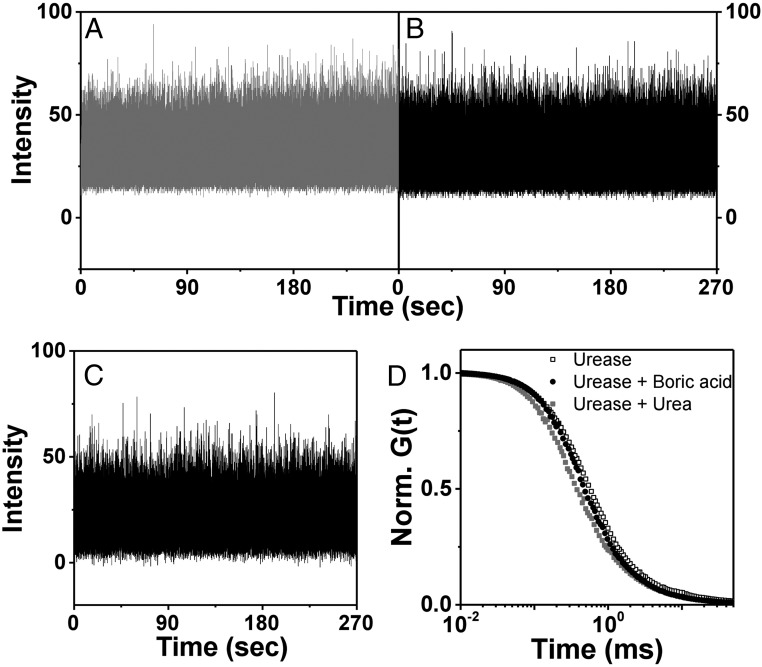
Representative fluorescence intensity is plotted against time in control experiments to search for pathological photophysics: (*A*) 10 nM urease labeled with Alexa 488 dye without substrate; (*B*) 10 nM urease labeled with Alexa 488 dye and 100 mM substrate (urea); (*C*) 10 nM urease labeled with Alexa 488 dye, 100 mM substrate (urea), and 100 mM inhibitor (boric acid). The respective intensity–intensity autocorrelation functions, normalized to unity at short times, are plotted against logarithmic time lag.

The possibility has been voiced that complex photophysics might interfere with clear-cut interpretation of FCS experiments (5, 6). In principle, substrates might quench fluorescence and the triplet lifetime might grow to become comparable with transit time through the observation window. However, for our experimental system, we find that the fluorescence lifetime was independent of substrate concentration over the biologically relevant regime, unlike quenching that is expected to depend on concentration. Furthermore, ballistic motion was observed not only in pure buffer but also in the presence of crowding agent that slowed mobility by a factor of 10. Quenching would be expected to have the same timescale regardless of the presence or absence of crowding agent, also making this argument unlikely. Indeed, the presence of 100 mM inhibitor decreased fluorescence intensity by ∼30%, but in the presence of substrate only, fluorescence intensity was unaffected by substrate concentration over the biologically relevant regime of substrate concentration (Fig. 5).

## Discussion

This is a noninertial low-Reynolds regime, and therefore the driving force must last through the boost’s stride, *l*_*B*_ ∼ 30–50 nm, and duration *τ*_*B*_ ∼ 1–10 μs. This rules out fast mechanisms, such as the active chemoacoustic pulse (4) and the passive binding-dependent viscoelastic moduli (21), with their picosecond timescales. Moreover, boosting the enzyme for 50 nm in 10 ps would require energies on the order of ∼10^6^ *k*_*B*_*T*. Likewise, cross-diffusion and phoresis forces cannot induce a long-range directional boost and anyhow are measured only at high concentrations, $c\gg K_{M}$. Superresolution fluorescence measurements have identified that ballistic motion of enzymes owing to the catalytic event is the likely origin of their enhanced diffusivity and spatiotemporal programming, but the physical mechanism driving the boost is not yet known. In the current absence of the needed angstrom-scale experimental information, one can only speculate and reason.

Internal protein motion may exhibit slow interdomain modes with periods longer than microsecond (11), so transfer of the chemically released energy into the slow mechanical spectrum of the protein is one possibility. It is unclear, however, whether this large-scale motion—considered critical to protein function (9–15, 60)—could boost the enzymes over ∼50-nm distances. Hinge-like swimming would require multiple strides to traverse such distances, and therefore is unlikely, but more elaborate swim strokes may be feasible (61). At any rate, swimming cannot explain the boosted motion of small organometallic catalysts (54).

Chemical reaction involves reshuffling of electrons when covalent bonds break and form. Speculatively, such charge rearrangement might induce significant electric forces at the catalytic site, despite strong screening by surrounding water. A requirement would be that the charged or polarized state be long-lived to sustain the boost for *τ*_*B*_ ∼ 1–10 μs. Once released, the products of chemical reaction diffuse angstroms away in a matter of picoseconds, and the presumed boosting state should therefore occur during the time the reaction progresses from the transition state to product release (62), scaling typically as the turnover time ∼0.1–10 ms. Direct measurements of such interactions will be a demanding experimental challenge, beyond the reach of the techniques employed so far.

Our experiments show that the enzymes investigated perform boosted directional motion, leading to antichemotactic self-organization in the biologically relevant concentration regime, $c∼K_{M}$ (1, 56, 57). Antichemotaxis may be a mechanism for homogenizing the production rate in enzymatic reactions by diverting an additional supply of enzymes to substrate-poor regions. This may be biologically functional for enzymes catalyzing reactions of central metabolism, such as the carbohydrate energy cycles. The core-metabolism enzymes turn over much more rapidly than average, and their motion should therefore be considerably enhanced, with consequent spatiotemporal organization optimizing production efficiency. More subtle programs could be achieved by combining diffusing enzymes and membrane-bound ones, serving as cytoplasmic nanopumps (63).

Single-molecule measurements of enzyme activity reveal widely distributed kinetic parameters (64), and antichemotaxis may be functionally useful by transporting faster enzymes in the population to domains of lower concentration. Some enzymes function as building blocks of larger, supramolecular complexes, which their boosting mechanism may assist to steer. The effects suggested here could be modified by the intricate geometry of the crowded milieu of the cell. A future direction in this field would therefore be in vitro measurement of boosted motion, testing the hypothesis that the current experiments carry biological function. The experimental consequences are anticipated to be most obvious for enzymes whose turnover rate is high, as in the experiments described and analyzed here.

## Methods

### The Boosted Enzyme Model.

In the low-Reynolds regime, $\text{Re}∼10^{-5},$ we can disregard inertia for timescales longer than the inertial time $\tau_{I}=m/\gamma ∼5\;\text{ps}$, where *m* is the enzyme’s mass and *γ* is its friction coefficient. The overdamped Langevin dynamics is simply the following:

$$\gamma v\left(t\right)=f_{T}\left(t\right)+f_{B}\left(t\right).$$ [8]

One stochastic force is the standard uncorrelated thermal force $f_{T}$ with

$$\langle f_{T,i}\left(t\right)\;f_{T,j}\left(t\prime \right)\rangle =2\gamma k_{B}T\delta_{ij}\delta \left(t-t\prime \right),$$ [9]

where *i* and *j* are the *x*, *y*, and *z* components. The second is the catalytically driven boost $f_{B}$ whose direction becomes uncorrelated at the rotational diffusion timescale $\tau_{R}$,

$$\langle f_{B,i}\left(t\right)f_{B,j}\left(t\prime \right)\rangle =\frac{1}{3}f_{B}^{2}\delta_{ij}\exp\left(-\frac{\left|t-t\prime \right|}{\tau_{R}}\right),$$ [10]

and whose amplitude $f_{B}$ is constant during a boost time $\tau_{B}$. The fluctuation–dissipation theorem (Green–Kubo relation) (29, 30) relates the diffusion coefficient $D_{E}$ to the sum over the velocity correlation function,

$$D_{E}=\frac{1}{3}\int_{0}^{\infty }dt\langle v\left(t\right)\cdot v\left(0\right)\rangle .$$ [11]

Using the Langevin Eq. **8**, we rewrite [**11**] as follows:

$$D_{E}=\frac{1}{3}\int_{0}^{\infty }dt\langle v\left(t\right)\cdot v\left(0\right)\rangle =\frac{1}{3\gamma^{2}}\int_{0}^{\infty }dt\left(\langle f_{B}\left(t\right)\cdot f_{B}\left(0\right)\rangle +\langle f_{T}\left(t\right)\cdot f_{T}\left(0\right)\rangle \right).$$ [12]

Without loss of generality, we assume a boost in the time range, $0\leq t\leq \tau_{B}$, where $\tau_{B}$ is the boost duration. We integrate over the typical duration between boosts, 0≤t≤1/V, where the MM catalysis rate is $V=k_{\text{cat}}c/\left(K_{M}+c\right)$ and $k_{\text{cat}}$ is the turnover number, $K_{M}$ is the MM constant, and *c* is the substrate concentration. When the boost ends $f_{B}$ vanishes so we multiply [**10**] by step functions $\langle f_{B,i}\left(t\right)\cdot f_{B,i}\left(t'\right)\rangle \theta \left(\tau_{B}-t\right)\theta \left(\tau_{B}-t'\right)$ and integrate Eq. **12** to obtain Eq. **1**. Integration over the boost duration, $0\leq t\leq \tau_{B}$, of the force correlation [**10**] yields the Debye function, typical of wormlike chains (31, 32), in the expression for enhanced diffusion $D_{E}$ in Eq. **1**. The integral is averaged over a typical time 1/V between catalytic events, which adds the factor *V* to $D_{E}$.

To obtain the MSD ${\langle r^{2}\rangle }_{\text{fast}}$ during the boost, we perform the integral

$$\begin{array}{l} {\langle r{\left(t\right)}^{2}\rangle }_{\text{fast}}=\int_{0}^{t}\int_{0}^{t}d\tau d\omega \langle v\left(\tau \right)\cdot v\left(\omega \right)\rangle \\ =\gamma^{-2}\int_{0}^{t}\int_{0}^{t}d\tau d\omega \left(\langle f_{B}\left(\tau \right)\cdot f_{B}\left(\omega \right)\rangle +\langle f_{T}\left(\tau \right)\cdot f_{T}\left(\omega \right)\rangle \right). \end{array}$$ [13]

With the correlation function of the forces used in [**12**], we obtain Eq. **3**.

### Fitting the Transit Times of Wormlike Trajectories.

The fitting procedure includes three steps. First, we fit the MSD of enzymes without substrate ${\langle r^{2}\rangle }_{0}$ to standard 3D Brownian motion. The beam width *w* is proportional to the MSD with a factor α∼o(1) stemming from the beam’s shape, $w^{2}=\alpha \langle r^{2}\rangle$. Hence, we fit the beam width and transit times as $w^{2}=\alpha 6D_{0}t_{w}=A\cdot t_{w}$ and extract the fit parameter *A*. The standard fitting procedure minimizes the sum of squared differences between the logarithms of the fit and the measurements. We take the logarithm since we have a curve that spans over an order of magnitude. From independent measurement of $D_{0}$ by FCS, we find the geometric factor, $\alpha =A/\left(6D_{0}\right)∼0.67$, indicating that the average transit path is ∼20% longer than the beam waist. The friction coefficient is found from Einstein’s relation, $\gamma =k_{B}T/D_{0}$. In the next stage, we fit the transit times of the fast component according to Eq. **3**, $w^{2}=A\cdot t_{w}+B\cdot \left[\exp\left(-t_{w}/\tau_{R}\right)-1+t_{w}/\tau_{R}\right]$, from which we obtain the rotational diffusion time $\tau_{R}$ and the fit parameter *B*. In the third step, we fit the slow component with its enhanced diffusivity, according to Eqs. **1** and **2**, $w^{2}=A\cdot t_{w}+B\cdot V\cdot \left[\exp\left(-\tau_{B}/\tau_{R}\right)-1+\tau_{B}/\tau_{R}\right]\cdot t_{w}$, yielding the boost time $\tau_{B}.$ In the latter formula, *V* is the catalysis rate of the whole enzyme complex, hexamer for urease (27) and tetramer for AChE. Finally, we extract the rest of the physical coefficients from the fit parameters: The boost force is $f_{B}=\left(\gamma /\tau_{R}\right)/\sqrt{B/\left(2\alpha \right)}$, from which we obtain the boost velocity $v_{B}=f_{B}/\gamma$ and length $l_{B}=v_{B}\tau_{B}$. The dissipated energy (in $k_{B}T$ units) is $b=l_{B}f_{B}/k_{B}T$, and the out-of-equilibrium parameter *p* is extracted from the proportion $p=\left(\tau_{R}/\tau_{B}\right)b$. The physical scales and dimensionless numbers extracted from the fits are listed in Table 1.

**Table 1.** Fit to wormlike trajectories in log scale

Enzyme	$f_{B}$, pN	$\ell_{B}$, nm	$\tau_{B}$, μs	b, *k*_*B*_*T*	p	$\tau_{R}$, μs	$v_{B}$, nm/μs
Urease	1.0	43	6.4	10	8.9	5.6	6.8
Urease (20% Ficoll)	1.1	14	19	3.6	11	57	0.8
AChE	0.4	35	17	3.2	8.8	46	2.1

The *R*^2^ coefficient is typically 0.98–0.99. As a control for the reliability of the fit, we compare the minimization of the sum of squares of the differences of the logarithms, the method used to extract the parameters in the text, to minimization of squares of the differences of the values themselves. The resulting parameters are listed in Table 2. The difference in the physical parameters between the logarithmic and linear methods is typically within a range of 20–30%. Importantly, the boost length $l_{B}$ and the nonequilibrium parameter *p* hardly change. On the other hand, the rotational timescale $\tau_{R}$ of urease changes by ∼50%, because the linear method optimizes the fit in the Fickian regime of the fast component curve. The timescale $\tau_{R}$ of AChE remains almost the same because most of the points are in the boosted region. However, since in this paper we focus on understanding the curved nondiffusive region, it is natural to use the logarithmic method as described in the text.

**Table 2.** Fit to wormlike trajectories in linear scale

Enzyme	$f_{B}$, pN	$\ell_{B}$, nm	$\tau_{B}$, μs	b, *k*_*B*_*T*	p	$\tau_{R}$, μs	$v_{B}$, nm/μs
Urease	1.3	47	5.3	14	8.0	2.9	8.9
Urease (20% Ficoll)	1.5	15	14	5.4	9.4	24	1.1
AChE	0.4	35	16	3.4	9.1	44	2.1

### Experimental.

The microfluidic chip with two sample inlets was fabricated from polydimethylsiloxane as described previously (7). First, the chip was flushed for 10 min to promote system equilibration including wetting of the chip walls, then flow of enzyme and buffer was initiated, measurements began 10 s after this time, and they were continued for 20–30 s. Regarding enzymes, urease from jack bean (>600,000 units/g; Sigma) was labeled at the amine residue with Alexa 488 in 150 mM phosphate buffer (pH 7.0) with added 2 µM urease and 40 µM fluorescent dye solution, stirred for 6 h at room temperature, and followed by extensive membrane dialysis (Amicon Ultra-4 centrifugal filter; Millipore) to remove free dye. Enzyme catalysis reactions were studied in buffer solution and also in solution to which 20% Ficoll 70 (Sigma) was added to produce viscosity 10 cP, in both cases in 150 mM phosphate buffer with pH adjusted to 7.2. Diffusion coefficients in the microfluidic chips were determined spot by spot, using standard confocal FCS by fitting intensity–intensity autocorrelation functions to the model of a single diffusion coefficient.

For urease inhibition using competitive inhibitors, boric acid (BioReagent Grade, >99.5%; Sigma) and acetic hydroxamic acid (98%; Sigma) were used. With boric acid, from 50 µM to 0.75 M boric acid and 1 µM urease were incubated for 30 min in 0.1 M PBS buffer (pH 7.0) at 35 °C. With acetic hydroxamic acid, mixtures of 1 µM urease in 0.1 M PBS buffer (pH 7.7) and concentration range from 50 µM to 2 M acetic hydroxamic acid were incubated at 35 °C for 1 h. This was added to buffer solution mixed with the substrate such that the final urease concentration was 10 nM.

The STED-FCS experiments (Leica TCS SP8X; Leica) used a 100× oil-immersion objective lens with N.A. of 1.4, an excitation wavelength of 488 nm, and depletion wavelength of 592 nm, with excitation at 80 MHz and a pulse width of 80 ps. Emitted fluorescence was collected using an avalanche photodiode (Micro Photon Devices; PicoQuant) through a 500- to 550-nm bandpass filter and recorded using a time-correlated single-photon counting detection unit (Picoharp 300; PicoQuant), which is integrated into the microscope and saves detected photons on the fly as data are acquired. The excitation laser and the depletion laser were superposed and the system was freshly realigned before each measurement. Using the microscope software (SymPhoTime; PicoQuant), this allows reconstruction of fluorescence lifetime decays as well as FCS data. From standard analysis of the intensity–intensity autocorrelations, the short time limit is approximately the inverse of the average number of dye molecules in the confocal volume, in our case ∼1.5 for 10 nM and 50 nm window sizes but ∼200 for 10 nM and 250 nm window sizes.
